# Public health and chronic low chlordecone exposures in Guadeloupe; Part 2: Health impacts, and benefits of prevention

**DOI:** 10.1186/s12940-016-0159-3

**Published:** 2016-07-19

**Authors:** Vincent Nedellec, Ari Rabl, William Dab

**Affiliations:** Director of Vincent Nedellec Conseil, 23, rue André Masséna–83000, Toulon, France; Retired from Ecole des Mines/ARMINES, Paris, Consultant on Environmental Impacts, 6 av. Faidherbe, 91440 Bures sur Yvette, France; Conservatoire National des Arts et Métiers (CNAM), Laboratoire MESuRS (Modélisation, épidémiologie et surveillance des risques sanitaires EA 4628), 292, rue Saint Martin–75141, Paris cedex 03, France

**Keywords:** Chlordecone, Low-dose, Threshold, Risk assessment, Non-mutagen agent, Endocrine disrupter, Risk management, Exposure reduction program

## Abstract

**Background:**

Inhabitants of Guadeloupe are chronically exposed to low doses of chlordecone via local food due to its past use in banana plantations. The corresponding health impacts have not been quantified. We develop a quantitative method and present the results in two articles: 1. Hazard identification, exposure-response functions, and exposure, 2. Health impacts, and benefits of a program to reduce the exposure of the population. Here is the second article.

**Methods:**

The exposure-response functions derived in Part 1 (for liver and prostate cancer, renal dysfunction and cognitive development) are combined with the exposure data to calculate the impacts. The corresponding costs are calculated via DALY’s and VOLY. A no-effect threshold is included via the marginal fraction of the collective exposure above the reference dose. The health benefits are the impacts in 2002 (before the exposure reduction program) minus the impacts in 2006 (since the program). They are compared to the costs, namely the public annual expenditures for reducing the population exposure.

**Results:**

Without threshold, estimated annual cases of liver cancer, prostate cancer and renal dysfunction are respectively 5.4, 2.8, 0.10 in 2002; and 2.0, 1.0, 0.04 in 2006. Annual IQ points lost (cognitive development) are respectively: 1 173 and 1 003. The annual cost of total impacts is 38.3 Million Euros (M€) in 2002 and 23.7 M€ in 2006. Comparing the benefit of 14.6 M€ with the 3.25 M€ spent for prevention, the program appears well justified. With threshold, the costs of the impacts are lower, respectively: 26.5 M€ in 2002 and 12.8 M€ in 2006, but the benefit is not very different: 13.7 M€.

**Conclusion:**

This is the first study that quantified chronic non genotoxic effects of chlordecone exposures in Guadeloupe. According to our results, preventive actions should be focused on pregnant women because of the high social cost of development impairment and also because their exposures decreased less rapidly than others. Prevention effort should be sustained as long as chlordecone remains in soils. Additional toxicological and epidemiological research would also be required for health endpoints that could not be taken into account (neurotoxicity of adults, autoimmune diseases and other developmental effects).

**Electronic supplementary material:**

The online version of this article (doi:10.1186/s12940-016-0159-3) contains supplementary material, which is available to authorized users.

## Background

Chlordecone has been used as pesticide for banana plantations. The inhabitants of the French West Indies are chronically exposed to low doses of chlordecone via local food. The corresponding public health issues have not been satisfactorily quantified because of the lack of an appropriate risk assessment method. Nonetheless, several million euros have been invested every year since 2003 for the reduction of exposure in Martinique and Guadeloupe [1–3]. Official investigations had revealed that 1.3 % of adults in Guadeloupe were exposed above the “no effect threshold dose” (the reference dose RfD) of 0.5 μg/kg/d [4]. Exposure to chlordecone seems more common in children 3 to 5 years old than in adults, with up to 18.5 % above RfD in Martinique [5]. These estimates of ingestion exposures are based on food consumption survey data matched with the results of chlordecone measurements in food made for the screening and control program. The main activities of the control program are to withdraw food from the market and water from the distribution system when chlordecone is above the limit values [6]. However, some polluted food from individual gardens could be eaten by the owners or the relatives and friends because they escape the market control system. It is very likely that chlordecone has also been used in other tropical countries, but no other studies have been carried out to assess its impacts.

The RfD is based on a chronic animal study that observed an increase in kidney damage (glomerulosclerosis) in female Wistar rats [7]. However, monitoring of poisoned workers manufacturing chlordecone in the USA (Hopewell and Baltimore) revealed no renal function impairment [8, 9]. In addition, a recent study showed that only mouse strains predisposed to auto immune diseases developed kidney lesions after ingesting chlordecone [10]. Accordingly, the hazards of chlordecone in people with exposure above this RfD are uncertain, and the results of exposure studies are difficult to translate into public health decisions. In particular, they do not provide answers to the following questions:

- What effect will occur in persons exposed above RfD: that observed at the lowest dose tested in animals, other effects known at some higher doses, or even the effect of another ubiquitous pollutant that is enhanced by chlordecone?
- If exposure exceeds the RfD what is the likelihood that an effect occurs?
- Are we sure that there will be no effect from exposures below RfD?

Several epidemiological studies have recently been carried out in Guadeloupe. The results show that chronic low exposure to chlordecone, mainly below the value of the RfD, is correlated with an increased risk of prostate cancer in men over 45 years [11] and with impaired neurobehavioral development in the young child [12, 13]. Other effects were investigated but were not significantly associated with low exposure to chlordecone: change in sperm quality [14, 15], and the rate of circulating hormones in men [16]. No studies looked at possible kidney damage, the critical effect found in laboratory animals [17, 18] nor at liver cancer which is significantly increased in a chronic animal study by the NCI [19]. The latter is used by the US-EPA to develop a cancer slope factor. In epidemiological studies exposure was estimated via blood chlordecone concentration (Cl-b).

Another concern for chlordecone health risk assessment is that we do not know sufficiently well the correspondence between Cl-b and the dose by ingestion expressed as mg/kg_BW_. First, an ad hoc study in Guadeloupe published in 2010 found that exposure data based on food consumption combined with food concentration of chlordecone are poorly correlated (*R2* = 0.20) with blood chlordecone [20]. Second, there was no PBPK (physiologically based pharmacokinetic) model available at the time of the literature search that could help to convert a dose by ingestion into a blood concentration of chlordecone.

Health risk assessment cannot be quantitative without a quantitative relationship between exposure and response. They are commonly available for carcinogenic effects but not for other effects that have a non-genotoxic mode of action. To quantify the public health impacts of chlordecone in Guadeloupe exposure-response functions (ERFs) are necessary. Following the recommendations of the Silver Book [21], we assume that non-genotoxic effects can occur at low doses if the mode of action has been found to be active at low doses and is shared by a frequent human disease or a ubiquitous chemical. In that case exposure-response functions, similar to those for genotoxic carcinogens, can be derived for non-genotoxic effects. Our objective is to develop a methodological framework based on: 1. the selection of potential hazards from low dose exposures; 2. deriving ERFs from human or animal data; 3. evaluation of health and economic impacts; 4. compare costs of the exposure control program with public health gains resulting from the decrease in population exposure. Because of their length, methods and results are described in two separate articles. Here is the second article whose objectives are: 1) to assess the health risks corresponding to chlordecone exposure in Guadeloupe before and after implementation of the exposure reduction public program, 2) estimate the health impacts and costs, 3) compare the health benefits (defined as the difference in impacts before and after preventive actions) and the costs of the program.. The overall objective is to carry out a cost-benefit analysis of the exposure control program that has been implemented in Guadeloupe since 2003, i.e. compare its cost with the benefit of reduced health impacts. Since the IOM report in 1981 [22], it is widely accepted that the cost of environment-related health effects should be consistently evaluated for decision making in public health, and a great deal of research has been carried out since then to develop and improve the required methods. Of particular importance are two major governmental projects, one in the EU, the other in the USA, that have become de facto guidelines [23, 24]. Here we apply the methods of ExternE for the impacts of chlordecone, with updated monetary values for mortality [25].

## Methods

### Results from Part 1

The methods used for identification of hazards at low doses of chlordecone and for derivation of the corresponding exposure-response functions (ERFs) were presented in Part 1. Briefly, with that methodology we found four possible effects at chronic low chlordecone dose: cancers, developmental impairment, hepatotoxicity, and neurotoxicity in adults. For neurotoxicity in adults the lack of a quantitative study precludes ERF derivation. With the traditional approach of public health agencies renal toxicity is considered as the critical effect; therefore we also derive an ERF for this effect. Two of these ERFs were derived from animal studies: kidney damage (ERF = 0.0022 mg/kg/d) [7] and liver cancer (ERF = 2.69 per mg/kg/day) [19]. The two others were derived from recent epidemiological studies: prostate cancer in men over the age of 44 years (ERF = 0.0019 mg/l_blood_) [11] and impairment of cognitive development in boys (ERF = −0.32 IQ_point_ per μg/l_cord-blood_) [12, 13]; for girls the associations with cognitive development were not significant. The four ERFs are based on absolute risk (or excess risk), they are used without the need to know the background incidence in the population. The cancer ERFs are adjusted for a standard lifetime exposure of 70 years.

Exposures of the Guadeloupe population are known thanks to blood chlordecone assays conducted for epidemiological studies. They have been described in Part 1. Briefly, the available data relate to two separate periods of time: before 2003, and after 2003 when preventive actions started. They are expressed in μg of chlordecone per liter of blood (μg/L_blood_). The quartiles used to describe the distribution of exposures in the population come from a review article [26].

For prostate cancers and cognitive development the ERFs are based on blood chlordecone; it will be marked by a subscript “b” added to the ERF and to the exposure “Exp”. The ERFs for liver cancer and kidney damage are based on ingested doses, marked by a subscript “i” for ERF and Exp. A conversion factor between ingestion dose and blood chlordecone was derived from the available pharmacokinetic data (see Part 1). Briefly, they showed a half-life of blood chlordecone in humans ranging from 63 to 192 days with an average of 127.5 days. The conversion factor (CF) obtained according to a biphasic (uptake/excretion) linear model is: CF_mea*n* =_ 0,064 (μg/kg/d)/(μg/L), with lower and upper limits CF_min._ = 0,043 (μg/kg/d)/(μg/L) and CF_max._ = 0,131 (μg/kg/d)/(μg/L).

### Health impact assessment

This begins by calculating the excess risk for chronic effects of chlordecone exposure with the following equations:

1 $$ \mathrm{E}\mathrm{R} = {\mathrm{ERF}}_{\mathrm{b}} \times {\mathrm{Exp}}_{\mathrm{b}} $$

ER = excess risk for a given exposure level (unitless)

ERF_b_ = exposure-response function based on blood chlordecone ((μg/L_blood_)^−1^)

Exp_b_ = exposure level based on blood chlordecone (μg/L_blood_)

2 $$ {\mathrm{ERF}}_{\mathrm{b}} = {\mathrm{ERF}}_{\mathrm{i}}/\mathrm{C}\mathrm{W} \times {\mathrm{CF}}_{\mathrm{i}/\mathrm{b}} $$

ERF_b_ = ERF based on blood chlordecone ((μg/L_blood_)^−1^)

ERF_i_ = ERF based on ingestion dose ((mg/kg/d)^−1^)

CW = conversion factor for mass units: 1000 μg/mg

CF_i/b_ = conversion factor between ingestion dose and blood chlordecone (μg/kg/d)/(μg/L)

Health impacts are defined as the number of cases due to population exposure. They are calculated for one year of exposure simply by multiplying the number of people at risk by the value of the excess risk:

3 $$ \mathrm{I} = \mathrm{E}\mathrm{R} \times \mathrm{n} $$

I = impact (number of cases)

ER = excess risk for a given exposure level (−)

*n* = number of people at risk

The impacts, being subsequently monetized with the value of a life year lost (VOLY), must be expressed as equivalent number of deaths, except for impairment of cognitive development for which the monetary value is based on the loss of lifetime income. The ERFs for liver cancer and for kidney damage are based on animal number of deaths in the studies. The one for prostate cancer is based on human incidence of new cases. The annual number of new cases of prostate cancer is very different from the annual number of deaths. The Guadeloupe data indicate a ratio of 4.7 between new cases and deaths [27]. Thus, the impact expressed as number of deaths from prostate cancer must be 4.7 times smaller than the impact estimated as number of new cases.

The ERF for liver cancer applies to all individuals in the population regardless of age; the ERF for prostate cancer only applies to men older than 44 years and the ERF for kidney damage only to women of all ages. The ERF for impaired cognitive development applies to male newborns.

Each of these population categories is divided into 5 exposure groups, characterized by the average interquartile value (Part 1). The first group includes individuals whose exposure is between 0 and the value of the analytical detection limit (DL). The second group includes individuals whose exposure is greater than the DL and lower than percentile 25 (P25). The proportion of individuals in this group is variable and is 25 % minus than the proportion of individuals in the first group. The third group includes the 25 % of the population whose exposure is between P25 and P50; the two last groups are analogous. The numbers of individuals in each population category come from national census data for Guadeloupe [28]. For the period before 2003, the reference year is 2002 and for the period after 2003, the reference year is the 2006. INSEE gives an annual growth rate of the population between 1999 and 2005 of 1.2 %. The figures for 2006 are extrapolated from 2005 with growth of 1.2 %. In Guadeloupe in 2002 the numbers in the population categories are approximately 437,000 persons (for liver cancer), 227,500 women (for kidney damage), 72,500 men over 44 years (for prostate cancer), and 3,750 newborn boys (for cognitive development). In 2006 the corresponding numbers are respectively 458,000; 239,500; 76,050; and 3,930.

### Consideration of a no effect threshold

A strong assumption of our main approach is to consider the effects of chlordecone without “no-effect threshold”. It is, however, interesting to see what happens if a threshold is introduced. The true collective no-effect threshold is not known. For illustration here we consider it equal to the reference value derived by the health and safety agencies: 0.5 μg/kg/day for adults. We derive a reference dose for effects on development, 0,165 μg/kg/day, as the average of two reference doses (RfD) found in the US-EPA toxicological profile of chlordecone regarding developmental effects [18]. One study shows, in Wistar rat exposed in utero via maternal diet, a reduced body weight gain 100 days after birth (RfD = 0.03 mg/kg/day; Squibb, 1982), the second shows in mice, with the same exposure pathway, a significantly reduced litter size (RfD = 0.3 mg/kg/day; Good, 1965). This threshold of 0.165 mg/kg/day applies to the exposure of pregnant women and not to the newborn.

A simple and rough way to introduce a threshold in the calculation of impacts would be to multiply the results obtained without threshold by the proportion of the population exposed above the threshold. However, regardless of the true form of the exposure response relationship, it is not the population fraction above threshold that matters, but rather the portion of the collective exposure that is above the threshold [29]. The collective exposure is the sum of e_i_ individual exposures of the population. A lognormal distribution has been found to be best general model for the distribution of environmental pollutant exposures [30]. The fraction of the collective exposure that is above the threshold (F_thr_) is calculated by means of the following equation:

4 $$ {\mathrm{F}}_{\mathrm{thr}}={\displaystyle {\sum}_{1={\mathrm{p}}_{thr}}^{\mathrm{p}}{\mathrm{e}}_1}/{\displaystyle {\sum}_{1=0}^{\mathrm{p}}{\mathrm{e}}_1} $$

F_thr =_ fraction of collective exposure that is above the threshold (−)

*p* = total population

p_thr_ = number of individuals with exposure ≤ threshold

e_i_ = exposure of individual i, in order of increasing i.

The impacts with a no effect threshold are obtained by multiplying the impacts without threshold by F_thr_:

5 $$ {\mathrm{I}}_{\mathrm{thr}} = \mathrm{I} \times {\mathrm{F}}_{\mathrm{thr}} $$

I_thr_ = impact with threshold (number of cases)

I = impact without threshold dose (number of cases)

F_thr =_ collective fraction of exposure above the threshold (−)

### Assessment of Benefits and Costs

For chronic risk the best approach may be based on disability-adjusted life years (DALY) and value of life year (VOLY) concepts [30, 31]. One DALY can be thought of as one lost year of “healthy” life. DALYs used here come from the WHO estimates for France during the years 2000 and 2012 [32, 33]. In our study, the reference year for all monetary values is 2006. Between 2000 and 2012 there has been some shift in DALY’s values. The observed trend is due to changes in incidence rates, mortality rates and age at death. To obtain the 2006 value we have made a simple interpolation between 2000 and 2012 values. This is possible because the WHO method has not changed between 2000 and 2012 [34]. DALY attributable to liver cancers, prostate cancers and kidney damage are respectively: 21.9 yr/case 16.5 yr/case and 26.1 yr/case.

Several methods exist for estimating the cost of mortality. The OECD recommends basing cost-benefit studies for public policy on contingent valuation surveys for the willingness-to-pay to avoid a premature death [35]. However, for the VOLY needed for most pollution impacts, in particular of chlordecone, not enough contingent valuation surveys have been carried out and their results are not sufficiently consistent. For that reason the guidelines for France published by the Commission chaired by Quinet [25] recommend that VOLY should be derived from the Value of Statistical Life for which reliable numbers have been provided by OECD [35]. We agree with the recommendations of Quinet. They imply a VOLY of 111 327 € in 2006, after adjustment for inflation. Knowing the number of DALY per case and the value of a VOLY one can simply estimate the cost of a case by multiplying the DALYs by VOLY. The cost of the impact I is calculated using the following equation:

6 $$ \mathrm{C}\mathrm{I} = \mathrm{I} \times \mathrm{DALY} \times \mathrm{VOLY} $$

CI = Cost of Impact (€/y)

I = impact with or without threshold dose (annual number of cases)

DALY = Disability Adjusted Life Year (y/cases)

VOLY = Value of Life Year (€/y)

The DALY approach is not used for impaired cognitive development, because there is no clear correlation between IQ level and life expectancy. Some studies have shown an inverse correlation between IQ and the risk of premature death (death before age 50), but the statistical association disappears if adjusted for the socio-economic level of the person [36, 37]. Conversely, in the same study, the correlation between socio-economic level and risk of premature death is not influenced by the level of IQ. On the other hand, North American studies on lead exposure of children have shown a strong link between loss of IQ points in childhood and loss of the lifetime income. The implied value of an IQ point in the North American studies was extrapolated to France to yield the cost of 1 lost IQ point as 17 363 €_2008_ (value in 2008) [38]. This value was used without modification to assess the economic impacts associated with mercury exposure in Europe [39]. So we assume the hypothesis that this value also applies to IQ points lost by chlordecone exposure. Given the inflation rate between 2006 and 2008 this value becomes: 16 837 €_2006_/IQ_point_. The unit costs of the four impacts included in our study are presented in Table 1.

**Table 1:** Unit values for the monetarization of health impacts

Health outcome	DALY^a^ (yr/case, France 2006)	VOLY^b^ (€_2006_)	Unit value = DALY × VOLY (€_2006_)
Liver cancer (per case)	21.9	111 327	2 441 030
Prostate cancer (per case)	16.5	111 327	1 835 523
Renal dysfunction (per case)	26.1	111 327	2 902 790
Cognitive development (per IQ point)	na	na	16 837

^a^Disability adjusted live year, from WHO, 2014 [32, 33]

^b^Value of Life Year, From Quinet [25], after adjustment for inflation

Na: not appropriate

The benefits of prevention can be estimated in physical terms, as number of cases avoided, or in monetary terms, as avoided costs, avoided referring to the difference made by the prevention program. The implementation of the prevention program starts in 2003 (Part 1). The ERF being linear, the number of cases prevented is directly proportional to the decrease in exposures. We estimate the benefits attributable to the program as the difference between the cost of impacts in 2002 (before 2003) and cost of impacts in 2006 (after 2003).

In 2012 the government published an evaluation of the exposure reduction program costs for the years 2008–2010 [40]. The program involves a wide variety of activities, such as monitoring of water wells and closure of polluted wells, monitoring of soils and limiting the planting of polluted soils, monitoring of food and removing polluted food from the market; the program also includes information campaigns, for farmers and homeowners about growing of food. It is not known how much exposure reduction is attributable to each of these actions. Annex 5 of the report shows the costs for the activities carried out in Martinique and Guadeloupe. The population of these two islands being roughly equivalent, we take the expenditures for Guadeloupe to be half of the total. The spending over three years was 20.7 million € or 6.9 million €/year and about 3.45 million €/year for Guadeloupe. Adjusted for inflation this value becomes: 3.25 million €_2006_/year for 2006.

### Uncertainties

As usual in this field, the models and parameters are more or less uncertain. A rigorous analysis of the uncertainty of the result would necessitate estimating the uncertainty distributions of all the parameters and models, and carrying out a Monte Carlo calculation to obtain the uncertainty distribution of the result. Such a task is even more difficult and uncertain than the calculation of central values and beyond the scope of this paper (see Chapter 11 of [30]).

However, one can obtain at least a rough first indication of the uncertainties by focusing on the most uncertain elements of the calculation, because elements with relatively small uncertainties have only a small effect on the overall uncertainty of the result. In our work the most uncertain elements are the exposure-response function (ERF), the assumption about a possible threshold, and the estimation of exposure (ingestion dose or blood chlordecone). For each of these we will show how much the results are changed by different assumptions: in Table 2 for the ERF, in Tables 3 and 4 for the threshold.

**Table 2:** The most uncertain parameters in the ERFs of chronic chlordecone effects

Health outcome	Most uncertain parameter	Unit	Mean value^a^	Low value^a^	High value^a^	Ratio high/low value
Liver cancer	CF_i/b_	(μg/kg/d)/(μg/L)	0.064	0.043	0.131	3.05
Prostate cancer	OR-1	(−)	0.77	0.21	1.58	7.52
Renal dysfunction	BMD_10-HED_	(mg/kg/d)^−1^	0.013	0.001	0.032	28.87
Cognitive development	IQ to ASQ equivalence	IQ_pt_/ASQ_pt_	1	0.2	2	10.00

CF_i/b_: conversion factor of ingestion dose to blood concentration of chlordecone. OR-1: difference of risk in exposed and reference populations. IQ to ASQ equivalence: IQ equivalence of 1 point of fine motor skill score in Age and Stage Questionnaire

^a^Taken from Part 1

**Table 3:** Health impacts, annual costs and benefits from reducing chlordecone exposures in Guadeloupe (Without threshold)

Health outcome	Main source of variation	Population at risk	Period of exposure measurement	*n*	Impact (mean estimate)	CI low (€_2006_)	CI mean (€_2006_)	CI high (€_2006_)	Proportion (mean estimate)
Liver cancer	Conversion from ingestion dose to blood chlordecone	All	<2003	436 968	5.4	8 821 000	13 129 000	26 874 000	34 %
			≥2003	458 465	2.0	3 274 000	4 873 000	9 974 000	21 %
			Annual benefit		−3.4	5 547 000	8 256 000	16 900 000	56 %
Prostate cancer	IC_95%_ of RR	Men > 44 years	<2003	72 566	2.8	1 405 000	5 152 000	10 572 000	13 %
			≥2003	76 049	1.0	490 000	1 797 000	3 687 000	8 %
			Annual benefit		−1.8	915 000	3 355 000	6 885 000	23 %
Renal dysfunction	BMD_10-HED_	Women	<2003	227 417	0.10	27 000	308 000	769 000	0.80 %
			≥2003	239 470	0.04	11 000	128 000	320 000	0.54 %
			Annual benefit		−0.06	16 000	180 000	449 000	1.23 %
Cognitive development	Equivalence between QI point and ASQ point	Male new born	<2003	3 747	1 173	3 949 000	19 745 000	39 489 000	52 %
			≥2003	3 929	1 003	3 377 000	16 887 000	33 773 000	71 %
			Annual benefit		−168	572 000	2 858 000	5 716 000	20 %
Total			<2003			14 202 000	38 334 000	77 704 000	100 %
			≥2003			7 152 000	23 685 000	47 754 000	100 %
			Annual benefit			7 050 000	14 649 000	29 950 000	100 %

*n* = population, 2002 census data for the first period of time and 2006 census data for second period. Impacts are expressed in number of death, except for cognitive development where the impacts are expressed in QI point lost. CI = Cost of Impacts (= DALY * VOLY)

Impact is the number of cases (Eq.3), except IQ points for Cognitive development

**Table 4:** Health impacts, annual costs and benefits from reducing chlordecone exposures in Guadeloupe (With threshold)

Health outcome	Main source of variation	Population at risk	Period of exposure measurement	*n*	F_thr_	Impact	CI low (€_2006_)	CI mean (€_2006_)	CI high (€_2006_)	Proportion (mean estimate)
Liver cancer	Conversion from ingestion dose to blood chlordecone	All	<2003	436 968	47 %	2.5	4 174 000	6 213 000	12 717 000	23 %
			≥2003	458 465	29 %	0.6	951 000	1 415 000	2 896 000	11 %
			Annual benefit			−2.0	3 223 000	4 798 000	9 821 000	35 %
Prostate cancer	IC_95%_ of RR	Men > 44 years	<2003	72 566	64 %	1.8	896 000	3 286 000	6 742 000	12 %
			≥2003	76 049	34 %	0.3	166 000	610 000	1 251 000	5 %
			Annual benefit			−1.5	730 000	2 676 000	5 491 000	20 %
Renal dysfunction	BMD_10-HED_	Women	<2003	227 417	31 %	0.033	8 000	95 000	237 000	0.36 %
			≥2003	239 470	24 %	0.011	3 000	31 000	77 000	0.24 %
			Annual benefit			−0.022	5 000	64 000	160 000	0.47 %
Cognitive development	Equivalence between QI point and ASQ point	Male new born	<2003	3 747	86 %	1005.1	3 384 000	16 922 000	33 844 000	64 %
			≥2003	3 929	64 %	652.4	2 155 000	10 779 000	21 557 000	84 %
			Annual benefit			−352.7	1 229 000	6 143 000	12 287 000	45 %
Total			<2003				8 462 000	26 516 000	53 540 000	100 %
			≥2003				3 275 000	12 835 000	25 781 000	100 %
			Annual benefit				5 187 000	13 681 000	27 759 000	100 %

*n* = population, 2002 census data for the first period of time and 2006 census data for second period. Fthr = fraction of collective exposure above threshold (for adults threshold = 0.5 μg/kg/d; for pregnant woman and effect on child development (cognitive) = 0.165 μg/kg/d). Impacts are expressed as number of deaths (mean estimate), except for cognitive development where the impacts are expressed in IQ point lost (mean estimate). CI = Cost of Impacts (= DALY * VOLY)

Impact is the number of cases (Eq. 3), except IQ points for Cognitive development

For liver cancer, the conversion from ingestion dose to blood chlordecone varies by a factor of 3. Since this factor is larger than the factor between the smallest and the largest BMD (=2.1; see Table 4 in Part 1) and between male and female best BMDs (Tables 3 and 4 in Part 1), we use a factor 3 to represent the uncertainty of the ERF of liver cancers. For prostate cancer the most uncertain parameter in the ERF is the 95 % confidence interval (95 % CI) around the risk differential (OR-1): central value = 0.77; lower limit = 0.21: and upper limit and 1.58. That is a factor 7.5 between the lowest value and the highest. For kidney damage the difference between the smallest and largest acceptable BMD_10-HED_ gives a factor of 29 (see Part 1). This factor being greater than that of the conversion factor, we take it as the uncertainty of the ERF kidney damage. For cognitive development the main uncertainty comes from the conversion of the ASQ scores (Age and Stage Questionnaire) measured at ages 7 and 18 months in the epidemiological study [12, 13] to IQ points, required for the monetary valuation. Such a conversion is obviously problematic because different aspects of cognitive abilities are measured. But there is no alternative because these ASQ scores are the only available quantitative data on the developmental neurotoxicity of chlordecone in humans. For the conversion factor we assume that 1 ASQ point = 1 IQ point as central value, with lower and upper bounds 0.2 IQ points and 2 IQ points, an interval that extends over a factor of ten. However, the reality of neurotoxic effects of chlordecone is not in doubt, because toxicological studies have identified relevant pathways for such effects in the body.

## Results

The health impacts without threshold, and the associated costs and benefits attributable to prevention are presented in Table 3 (details in Additional file 1). The benefits are significant, in physical and in monetary terms. For instance, the number of deaths from liver cancer before 2003 is estimated at 5.4/yr and after 2003 at 2.0/yr. That is 3.4 deaths/yr avoided by reducing the exposure. Before 2003, the total cost of health impacts (see Table 3) amounted to 38.3 million €_2006_/yr (denoted by M€/yr) (range from 14.2 to 77.7). Before 2003, impacts on cognitive development are the most important with 52 % of the total annual cost, followed by liver cancer (34 %), prostate cancer (13 %) and kidney damage (0.8 %). After 2003, the total cost of impacts is reduced to 23.7 M€/yr (from 7.2 to 47.8). The part of cognitive development in the total cost after 2003 increases to 71 %. This indicates a smaller decrease of in utero exposures compared to the decrease of others population exposures. Overall, the benefits due to the reduction of exposures are estimated at 14.6 M€/yr (from 7.0 to 30.0 M€/yr). The percentages of the total benefits are: liver cancer 56 %, cognitive development 23 %, prostate cancer 20 % and renal disease 1.2 %. Thus, with this calculation the annual total benefits are 4.5 times higher than annual cost of prevention (3.25 M€/yr).

Since cognitive development is the most uncertain impact, we note that if it were omitted entirely from the analysis, the benefit would be reduced from 14.6 M€/yr to 11.8 M€/yr, still 3.6 times as large as the cost.

Results obtained with threshold are presented in Table 4 (details in Additional file 1). The collective exposure fractions above threshold (F_thr_) in adults are higher (31 to 64 %) in the period before 2003 than after (24 to 34 %). They are also higher in men, respectively before and after 2003: 64 % and 34 %, than in women respectively 31 % and 24 %. However, they have dropped less in women than in men. The no-effect threshold dose for newborns being lower (0.165 versus 0.5 μg/kg/d), the F_thr_ are significantly higher. The decrease in F_thr_ for newborns after 2003 is lower than in men. The cost of impacts before 2003 totaled 26.5 M€/yr (from 8.5 to 53.5), about 69 % of the cost of impacts without threshold. The effects on cognitive development are the most important with 64 % of the total annual cost, followed by liver cancer (23 %), prostate cancer (12 %) and kidney damage (0.4 %). After 2003, the cost of impacts with threshold amounted to 12.8 M€/yr (from 3.3 to 25.8), about 54 % of the cost of impacts without threshold. The part of impaired cognitive development increases for the same reason as above. Overall, the benefits due to the reduction of exposures are estimated at 13.7 M€/yr (from 5.2 to 27.8). They are very close to the benefits calculated without threshold. They rank as follows: cognitive development 45 %, liver cancer 35 %, prostate cancer 20 % and renal disease 0.5 %. Thus, the annual total benefit estimated with a threshold dose is 4.2 times higher than the annual cost of prevention (3.25 M€/yr).

## Discussion

We have implemented the recommendations of the Silver Book of the National Research Council of the USA [21] and obtained exposure-response functions that make it possible to quantify the health risks of chlordecone, both carcinogenic and non-carcinogenic. We have calculated the cost of these health impacts using the methods of environmental economics, with specific monetary values recommended by Pichery [38] and Quinet [25]. We have calculated these costs before and after the implementation of the exposure reduction program. Thus we have been able to compare the benefit of this program with its cost (as reported in [40]). We find that the exposure reduction program is very well justified, whether or not one includes a no-effect threshold. The benefit is at least four times larger than the cost: the program appears well justified.

The methodology is general and can be used for other environmental pollutants.

One may, however, wonder if the uncertainties could invalidate the conclusion. There are two kinds of uncertainty, one related to measurement error and one related to lack of knowledge. The uncertainties of all the variables and assumptions in our model combine to determine the uncertainty of the results. A rigorous analysis would require Monte Carlo calculations, see for instance Chapter 11 of Rabl 2014 [30]. Here we opted for a much simpler approach, taking into account only the factor that is the most uncertain in the ERF: the OR for risk of prostate cancer, the ingestion dose to blood chlordecone (CF_i/b_) conversion factor for liver cancer, the BMD_10-HED_ for kidney damage, and finally the equivalence between IQ points and ASQ points for impaired cognitive development (see Table 2). We also consider that the measurement errors are smaller and included in the natural variation of the parameters. For example, the measurement error in blood chlordecone concentrations is less than 20 % (see appendix in Multigner et. al. 2010), whereas the range of measured values is a factor of 177 in the prostate cancer study (0.24 to 44.4 μg/l). Hence, this range represents very well the combination of measurement error and natural variability. The variability of exposures is taken into account by decomposition into groups corresponding to exposure quartiles. This option has been preferred to the use of a central value (average or median) with confidence interval because of the very skewed shape of the distribution of exposures. The costs are framed by low and high estimates representing the variability of the most variable parameter in the model.

To explain the uncertainties related to the lack of knowledge, recall that our main model has six parameters: the ERF, the conversion factor of ingestion dose to blood concentration, exposures, the number of people at risk, the DALY values and the VOLY value. The alternative calculations with threshold have a further parameter: the fraction of collective exposure above the threshold (F_thr_). Uncertainties related to ERF were discussed in the previous article (Part 1). Briefly, they contain neither safety factor nor uncertainty factor. Their points of departure (POD) are the central estimate of the risk indicators in the original studies. When multiple POD are possible, we do not choose one that will give the strongest slope, but the average POD. This makes it unlikely to overestimate risk in the range of known exposures. However, the linear extrapolation from the POD to the origin, may overestimate risk if the true shape of the dose–response relationship is sub-linear or has a no-effect threshold. The case of a potential threshold is discussed later. Concerning a sub-linear form, an overestimate is unlikely for prostate cancer because the ERF retained here derives from the most exposed group. The ERFs from the two lower exposed groups would be much higher (see Part 1 Table 1). As shown in Figure 1 of Part 1, the regression of the three ERFs is an inverted hockey stick curve, clearly supra-linear. This kind of exposure-response relationship is known in other endocrine disruptors [41]. Likewise we can assume that kidney damage and reduced cognitive development, two other effects of chlordecone mediated by endocrine mechanisms are in the same situation. For liver cancer the only available point of comparison is the cancer slope factor (Sf) derived by the US-EPA from the same study that we have used to derive our ERF. The US-EPA slope factor is 10 (mg:kg/g)^−1^, 3.7 times higher than our ERF of 2,692 (mg/kg/d)^−1^. This slope factor would estimate 3.7 times more risk and impacts. Hence, the uncertainty conveyed by our ERFs would imply an underestimation of the risks.

Uncertainties related to exposure estimations via blood chlordecone measurements have been described in Part 1. Briefly: the age groups of men before and after 2003 do not overlap (20–44 years before 2003 and > 44 years after 2003), and those of women are limited to the range 17–45 years. Newborn exposure data include girls and boys. The numbers of individuals are small and mostly smaller before 2003 than after. It is not possible to know in which direction these uncertainties affect the quantitative results. However the age group difference for men before and after 2003 may explain why their exposures have decreased more than for women and their children. That would imply that men younger than 44 years working outside the agricultural sector are more exposed than those over 44 years.

The population data come from INSEE, the French official census institution [28]. Their uncertainties are insignificant compared to the other uncertainties.

In our calculation we used blood chlordecone concentrations because they seem to better reflect actual exposures of the population than ingestion doses which have been shown to be poorly correlated with blood chlordecone [20]. However, important work of estimating ingestion doses has been produced by the French Agency for Environmental and Health Safety [6, 42, 43]. It would be therefore interesting to compare the results obtained with the blood chlordecone (our assessment) to results that would be obtained with the ingestion dose data by following the same procedure. Unfortunately, there are no ingestion dose data before 2005. The evaluation of benefits attributable to prevention therefore is not possible with these data. Moreover the assessment of impacts on cognitive development is not possible since by definition there is no ingestion dose in utero.

The concept of DALYs has been used since the 1990s by WHO to measure the global burden of disease. This indicator is widely recognized and used in health economic studies. The DALY/case values used here come from French statistics published by WHO [32, 33]. It includes two components: the years of life lost because of premature death and an adjustment for the years lived with the disease. Medical expenses, loss of productivity and insurance payments are not taken into account. This has the effect of underestimating the total cost, especially in the case of prostate cancer, because the annual number of incident cases of this disease is generally greater than the annual number of deaths.

The effects of chlordecone on cognitive development yield the highest cost contribution. The ERF is 0.32 IQ points lost per μg/l of cord blood chlordecone. By comparison the ERF for mercury is 4 times higher with 1.395 IQ points lost per μg/l in cord blood mercury [39]. The one for lead is less directly comparable since it is related to blood lead levels of children several years old, not newborns; it is 3.9 IQ points lost per ug/l for exposures between 24 and 100 μg/l [38]. Per μg/l in blood, chlordecone is less toxic for cognitive development than mercury and lead. In the original study on chlordecone, mercury and lead were also measured in cord blood along with other chemicals: PCB153 (polychlorinated biphenyl congener 153), p, p-DDE (dichlorodiphenyl Dichloroethylene) and DHA (docosahexaenoic acid). If mercury and lead had been found to be correlated with ASQ score for fine motor skills, it would have yielded a direct link between that score and IQ points. But with the exception of PCB153 and chlordecone none of these chemicals was correlated with the ASQ score for fine motor skills (with a p <0.2) [13]. Even though this may raise doubts about the equivalence between IQ point at 7 years and fine motor skills measured at 18 months by the ASQ score, a lack of such correlation for lead and mercury does not imply that chlordecone will not be correlated with IQ score at 7 years. Its low-dose toxicity for the nervous system has been shown by mechanistic studies (see Part 1). Today this uncertainty is difficult to reduce. Our assessment uses the available and relevant data and our results should be updated when new knowledge emerges.

The estimated fraction of collective exposure is uncertain because the means and standard deviations of blood chlordecone concentrations were not published in the original articles (except Multigner 2006). We used the weighted average of values of each exposure groups defined for each population category (see Part 1, Table 7). For example, the average blood chlordecone concentration for men 20–45 years before 2003 obtained by our weighted average is 7.1 μg/l. This value is close to the geometric mean published by the authors 8.4 μg/l [14], the only comparison point available. Standard deviations are determined by successive iterations to fit a log normal distribution to the values of known exposure quartiles. Only the use of original individual data would have reduced this uncertainty, but unfortunately they are not accessible. However, the impacts and costs with threshold are not very different from those without threshold, and the gains resulting from preventive actions are almost the same. This can be explained by the fact that the F_thr_ of newborns is much higher than the women’s F_thr_ because the threshold for newborns is lower, together with the very high cost contribution of cognitive development. Overall, the inclusion of a no-effect threshold reduces the costs of the health impacts by a factor 0.7 before and by a factor 0.54 after the intervention. These results seem specific to the particular distribution of measured blood chlordecone concentrations. Before 2003 over 50 % of the impacts are in the most exposed group (Group 5) regardless of the effect considered (see Additional file 1 tables A to J). This proportion rises to over 80 % of impacts after 2003.

The costs of the exposure reduction program come from the balance sheet of the 1st Chlordecone Plan during the period 2008–2010 [40]. We do not have data for 2003–2008, and we considered that the annual expenses were the same as for 2008–2010, with adjustment for inflation. Nevertheless, these costs are probably underestimated because, for example, the work of the civil service and local authorities dealing with this problem is not included.

Our comparison of costs and benefits indicates that the health benefits far outweigh the costs of the prevention program, whether evaluated with or without threshold. Kidney damage accounts for only 0.5 to 1 % of total health benefits and excluding it would not significantly change the conclusion. The same holds for cognitive development impacts: they account only for around 20 % of the total benefits due to the prevention program, and omitting them would not make the benefits smaller than the cost.

Not all prevention actions have the same efficacy, and it would be desirable to know the costs and benefits of individual actions. Unfortunately there are no data for that; the program can only be evaluated as a package.

## Conclusion

This work presents a double originality. This is the first quantitative health risk assessment with low dose exposure-response functions derived for a non-mutagenic chemical, an approach increasingly recommended for endocrine disrupters. It is also the first work to monetize health impacts of chlordecone. Our approach provides valuable information for public health decision making. Its flexibility allows comparing basic assumptions such as the existence of a no-effect threshold. It also allows a comparison of the cost of preventive actions with the corresponding health benefits. Finally the results facilitate the prioritization of health safety actions. For example, according to our results, pregnant women should be targeted by preventive actions because of the high social cost of development impairment and also because exposures of mothers and newborns are lowered less rapidly than others. Our quantitative approach is generally recommended for environmental factors to which populations are already exposed.

Actions for reducing chlordecone exposure in Guadeloupe seem effective and appear justified by their benefits, whether or not one assumes a no-effect threshold. The persistence of chlordecone in soil calls for continuing these actions in the long term. Determining the level of expenditures for the optimal reduction of exposures would necessitate more detailed assessments than we have presented. As a first approximation, maintaining the commitments of the first Chlordecone Plan appears to be a good policy to prevent exposures from rising again to levels existing before 2003. Repetitive monitoring of blood chlordecone concentrations in a representative population sample would be very useful for the management and assessment of these actions. The monitoring of food should be maintained in order to keep contaminated food out of distribution channels, but the utility of such data (ingestion dose) for risk assessment is doubtful in the case of chlordecone.

Epidemiological surveys would be needed to study the role of chlordecone in effects that have not been taken into account here for lack of knowledge about exposure-response relationships (neurotoxicity, autoimmune diseases, and other developmental effects). Toxicological knowledge available about chlordecone is old and mainly limited to acute effects. It would be useful to provide mechanistic studies on these “orphan” effects before implementing epidemiological studies. Finally, an extended follow-up of children in the TIMOUN cohort would allow reducing the uncertainty about the effect of chlordecone on cognitive development.

When making an assessment for environmental factors to which populations are already exposed, focusing only on the so called « critical effect » of a chemical can dramatically underestimate the total impacts and health costs. Another source of strong underestimation is the use of average values instead of detailed exposure distributions. The quantitative approach that we propose for non-genotoxic effects allows to avoid these biases.

## Abbreviations

ASQ, Age and stage questionnaire; BMD, Benchmark dose; BMD_10_, Benchmark dose for a 10 % excess risk; BMD_HED_, Benchmark dose expressed as Human Equivalent Dose; CF, Conversion Factor; DALY, Disability-Adjusted Life Years; DL, (analytical) Limit of Detection; ERF, Exposure-Response Function; F_thr_, cumulative fraction of exposure above the threshold; INSEE, Institut National de la Statistique et des Etudes Economiques (National Institute for Statistical and Economic studies); IQ, intelligence quotient; M€, million euros; MOA, Mode of action; OECD, Organization for Economic Cooperation and Development; OR, odds ratio; P25, P50, Px, Percentile 25, percentile 50, Percentile x; POD, point of departure; RfD, reference dose; RR, Relative risk; SLE, Systemic Lupus Erythematosus; US-EPA, United State Environmental Protection Agency; VOLY, Value Of Life Year; WHO, World Health Organization

## Additional file

Additional file 1:
**Table A**: Annual impacts and costs of liver cancer in Men exposed to chlordecone in Guadeloupe before 2003. **Table B**: Annual impacts and costs of liver cancer in Men exposed to chlordecone in Guadeloupe after 2003. **Table C**: Annual impacts and costs of liver cancer in Women exposed to chlordecone in Guadeloupe before 2003. **Table D**: Annual impacts and costs of liver cancer in Women exposed to chlordecone in Guadeloupe after 2003. **Table E**: Annual impacts and costs of prostate cancer in Men > 44 y exposed to chlordecone in Guadeloupe before 2003. **Table F**: Annual impacts and costs of prostate cancer in Men > 44 y exposed to chlordecone in Guadeloupe after 2003. **Table G**: Annual impacts and costs of renal dysfunction in Women exposed to chlordecone in Guadeloupe before 2003. **Table H**: Annual impacts and costs of renal dysfunction in Women exposed to chlordecone in Guadeloupe after 2003. **Table I**: Annual impacts and costs of cognitive development in boys born to Women exposed to chlordecone in Guadeloupe before 2003. **Table J**: Annual impacts and costs of cognitive development in boys born to Women exposed to chlordecone in Guadeloupe after 2003. (DOCX 122 kb)
