# Supplementary material for: Public health and chronic low chlordecone exposures in Guadeloupe; Part 2: Health impacts, and benefits of prevention
Source: Environ Health. 2016 Jul 19;15:78. doi: 10.1186/s12940-016-0159-3 (PMC4950608; doi:10.1186/s12940-016-0159-3)
Supplement: Additional file 1: — Table A: Annual impacts and costs of liver cancer in Men exposed to chlordecone in Guadeloupe before 2003. Table B: Annual impacts and costs of liver cancer in Men exposed to chlordecone in Guadeloupe after 2003. Table C: Annual impacts and costs of liver cancer in Women exposed to chlordecone in Guadeloupe before 2003. Table D: Annual impacts and costs of liver cancer in Women exposed to chlordecone in Guadeloupe after 2003. Table E: Annual impacts and costs of prostate cancer in Men > 44 y exposed to chlordecone in Guadeloupe before 2003. Table F: Annual impacts and costs of prostate cancer in Men > 44 y exposed to chlordecone in Guadeloupe after 2003. Table G: Annual impacts and costs of renal dysfunction in Women exposed to chlordecone in Guadeloupe before 2003. Table H: Annual impacts and costs of renal dysfunction in Women exposed to chlordecone in Guadeloupe after 2003. Table I: Annual impacts and costs of cognitive development in boys born to Women exposed to chlordecone in Guadeloupe before 2003. Table J: Annual impacts and costs of cognitive development in boys born to Women exposed to chlordecone in Guadeloupe after 2003. (DOCX 122 kb) [file 12940_2016_159_MOESM1_ESM.docx]

**Public health safety and chronic low chlordecone exposures in Guadeloupe. Part 2: Health impacts, and benefits of prevention.**

**Vincent Nedellec^1^, Ari Rabl^2^, William Dab^3^.**

**Additional file 1**

**List of tables**

[Table A: annual impacts and costs of liver cancer in Men exposed to chlordecone in Guadeloupe before 2003 2](#_Toc431989848)

[Table B : annual impacts and costs of liver cancer in Men exposed to chlordecone in Guadeloupe after 2003 3](#_Toc431989849)

[Table C : annual impacts and costs of liver cancer in Women exposed to chlordecone in Guadeloupe before 2003 4](#_Toc431989850)

[Table D : annual impacts and costs of liver cancer in Women exposed to chlordecone in Guadeloupe after 2003 5](#_Toc431989851)

[Table E : annual impacts and costs of prostate cancer in Men > 44 yr exposed to chlordecone in Guadeloupe before 2003 6](#_Toc431989852)

[Table F : annual impacts and costs of prostate cancer in Men > 44 yr exposed to chlordecone in Guadeloupe after 2003 7](#_Toc431989853)

[Table G : annual impacts and costs of renal dysfunction in Women exposed to chlordecone in Guadeloupe before 2003 8](#_Toc431989854)

[Table H : annual impacts and costs of renal dysfunction in Women exposed to chlordecone in Guadeloupe after 2003 9](#_Toc431989855)

[Table I : annual impacts and costs of cognitive development in boys born to Women exposed to chlordecone in Guadeloupe before 2003 10](#_Toc431989856)

[Table J : annual impacts and costs of cognitive development in boys born to Women exposed to chlordecone in Guadeloupe after 2003 11](#_Toc431989857)

Table A: Annual impacts and costs of liver cancer in Men exposed to chlordecone in Guadeloupe before 2003

| **Parameters** | **source** | **unit** | **Group 1** | **Group 2** | **Group 3** | **Group 4** | **Group 5** | **∑** |
| --- | --- | --- | --- | --- | --- | --- | --- | --- |
| ERF (lifetime exposure, absolute risk) | cf. article 1 | (mg/kg/d)^-1^ | 2.69 | 2.69 | 2.69 | 2.69 | 2.69 | na |
| CW (change in weight unit) | na | µg/mg | 1000 | 1000 | 1000 | 1000 | 1000 | na |
| CF_e/i_ | cf. article 1 | [µg/kg/d]/[µg/l_blood_] | 0.06 | 0.06 | 0.06 | 0.06 | 0.06 | na |
| Standard human lifetime | USEPA, 1988 [1] | yr | 70 | 70 | 70 | 70 | 70 | na |
| ERF (annual exposure, absolute risk) | equation 2 | (µg/l_blood_)^-1^ per year | 2.5E-06 | 2.5E-06 | 2.5E-06 | 2.5E-06 | 2.5E-06 | na |
| Exposure | Guldner, 2011 [2] | µg/l_blood_ | 1.06 | 1.56 | 3.90 | 7.25 | 14.79 | na |
| Excess Risk | equation 1 | (-) | 2.6E-06 | 3.9E-06 | 9.6E-06 | 1.8E-05 | 3.6E-05 | na |
| Exposure distribution | Guldner, 2011 [2] | (-) | 9% | 16% | 25% | 25% | 25% | 100% |
| Population | INSEE 2002 | n | 18 860 | 33 528 | 52 388 | 52 388 | 52 388 | 209 551 |
| Impacts | equation 3 | case | 0.05 | 0.13 | 0.50 | 0.93 | 1.91 | 3.52 |
| DALY | WHO 2014a [3] | yr/case | 21.93 | 21.93 | 21.93 | 21.93 | 21.93 | na |
| VOLY | Quinet, 2013 [4] | €_2006_/yr | 111 327 | 111 327 | 111 327 | 111 327 | 111 327 | na |
| **Cost of Impacts (CI)** | **equation 5** | €_2006_ | **120 573** | **315 136** | **1 227 508** | **2 281 906** | **4 654 619** | **8 599 743** |
| Proportion of the total cost | Calculated | % | 1% | 4% | 14% | 27% | 54% | 100% |
|  |  |  | **Mean estimate** | **Low estimate** | **High estimate** |  |  |  |
| **CI without threshold** |  | €_2006_ | **8 599 743** | **5 777 952** | **17 602 599** |  |  |  |
| **F_thr_** | equation 4 | (-) | **65.1%** | **65.1%** | **65.1%** |  |  |  |
| **CI with threshold** | CI × F_thr_ | €_2006_ | **5 596 337** | **3 760 039** | **11 455 003** |  |  |  |

na: not appropriate. F_thr_: Fraction of collective exposure above threshold. ∑ = the sum of results of group 1 to group5. The results highlighted in green are shown in tables of article 2.

Table B : Annual impacts and costs of liver cancer in Men exposed to chlordecone in Guadeloupe after 2003

| **Parameters** | **source** | **unit** | **Group 1**  **mean estimate** | **Group 2**  **mean estimate** | **Group 3**  **mean estimate** | **Group 4**  **mean estimate** | **Group 5**  **mean estimate** | **∑** |
| --- | --- | --- | --- | --- | --- | --- | --- | --- |
| ERF (lifetime exposure, absolute risk) | cf. article 1 | (mg/kg/d)^-1^ | 2.69 | 2.69 | 2.69 | 2.69 | 2.69 | na |
| CW (change in weight unit | na | µg/mg | 1000 | 1000 | 1000 | 1000 | 1000 | na |
| CF_e/i_ | cf. article 1 | [µg/kg/d]/[µg/l_blood_] | 0.06 | 0.06 | 0.06 | 0.06 | 0.06 | na |
| Standard human lifetime | USEPA, 1988 [1] | yr | 70 | 70 | 70 | 70 | 70 | na |
| ERF (annual exposure, absolute risk) | equation 2 | (µg/l_blood_)^-1^ per year | 2.5E-06 | 2.5E-06 | 2.5E-06 | 2.5E-06 | 2.5E-06 | na |
| Exposure | Guldner, 2011 [2] | µg/l_blood_ | 0.17 | 0.22 | 0.40 | 0.90 | 7.60 | na |
| Excess Risk | equation 1 | (-) | 4.1E-07 | 5.5E-07 | 9.8E-07 | 2.2E-06 | 1.9E-05 | na |
| Exposure distribution | Guldner, 2011 [2] | (-) | 13% | 12% | 25% | 25% | 25% | 100% |
| Population | INSEE 2002 | n | 29 126 | 25 623 | 54 749 | 54 749 | 54 749 | 218 996 |
| Impacts | equation 3 | case | 0.0120 | 0.014 | 0.054 | 0.12 | 1.02 | 1.23 |
| DALY | WHO 2014a [3] | yr | 21.93 | 21.93 | 21.93 | 21.93 | 21.93 | na |
| VOLY | Quinet, 2013 [4] | €_2006_/yr | 111 327 | 111 327 | 111 327 | 111 327 | 111 327 | na |
| **Cost of Impacts (CI)** | **equation 5** | €_2006_ | **29 267** | **34 482** | **131 573** | **296 039** | **2 499 016** | **2 990 376** |
| Proportion of the total cost | Calculated | % | 1% | 1% | 4% | 10% | 84% | 100% |
|  |  |  | **Mean estimate** | **Low estimate** | **High estimate** |  |  |  |
| **CI without threshold** |  | €_2006_ | **2 990 376** | **2 009 159** | **6 120 926** |  |  |  |
| **F_thr_** | equation 4 | (-) | **32.6%** | **32.6%** | **32.6%** |  |  |  |
| **CI with threshold** | CI × F_thr_ | €_2006_ | **975 514** | **655 423** | **1 996 755** |  |  |  |

na: not appropriate. F_thr_: Fraction of collective exposure above threshold. ∑ = the sum of results of group 1 to group5. The results highlighted in green are shown in tables of article 2.

Table C : Annual impacts and costs of liver cancer in Women exposed to chlordecone in Guadeloupe before 2003

| **Parameters** | **source** | **unit** | **Group 1**  **mean estimate** | **Group 2**  **mean estimate** | **Group 3**  **mean estimate** | **Group 4**  **mean estimate** | **Group 5**  **mean estimate** | **∑** |
| --- | --- | --- | --- | --- | --- | --- | --- | --- |
| ERF (lifetime exposure, absolute risk) | cf. article 1 | (mg/kg/d)^-1^ | 2.69 | 2.69 | 2.69 | 2.69 | 2.69 | na |
| CW (change in weight unit | na | µg/mg | 1000 | 1000 | 1000 | 1000 | 1000 | na |
| CF_e/i_ | cf. article 1 | [µg/kg/d]/[µg/l_blood_] | 0.06 | 0.06 | 0.06 | 0.06 | 0.06 | na |
| Standard human lifetime | USEPA, 1988 [1] | yr | 70 | 70 | 70 | 70 | 70 | na |
| ERF (annual exposure, absolute risk) | equation 2 | (µg/l_blood_)^-1^ per year | 2.5E-06 | 2.5E-06 | 2.5E-06 | 2.5E-06 | 2.5E-06 | na |
| Exposure | Guldner, 2011 [2] | µg/l_blood_ | 0.32 | 0.62 | 1.70 | 3.05 | 8.05 | na |
| Excess Risk | equation 1 | (-) | 7.9E-07 | 1.5E-06 | 4.2E-06 | 7.5E-06 | 2.0E-05 | na |
| Exposure distribution | Guldner, 2011 [2] | (-) | 13% | 12% | 25% | 25% | 25% | 100% |
| Population | INSEE 2002 | n | 29 564 | 27 290 | 56 854 | 56 854 | 56 854 | 227 417 |
| Impacts | equation 3 | case | 0.02 | 0.04 | 0.24 | 0.43 | 1.13 | 1.86 |
| DALY | WHO 2014a [3] | yr | 21.93 | 21.93 | 21.93 | 21.93 | 21.93 | na |
| VOLY | Quinet, 2013 [4] | €_2006_/yr | 111 327 | 111 327 | 111 327 | 111 327 | 111 327 | na |
| **Cost of Impacts (CI)** | **equation 5** | €_2006_ | **56 929** | **101 682** | **580 687** | **1 041 820** | **2 748 396** | **4 529 513** |
| Proportion of the total cost | Calculated | % | 1% | 2% | 13% | 23% | 61% | 100% |
|  |  |  | **Mean estimate** | **Low estimate** | **High estimate** |  |  |  |
| **CI without threshold** |  | €_2006_ | **4 529 513** | **3 043 267** | **9 271 348** |  |  |  |
| **F_thr_** | equation 4 | (-) | **34.5%** | **34.5%** | **34.5%** |  |  |  |
| **CI with threshold** | CI × F_thr_ | €_2006_ | **1 564 327** | **1 051 032** | **3 201 982** |  |  |  |

na: not appropriate. F_thr_: Fraction of collective exposure above threshold. ∑ = the sum of results of group 1 to group5. The results highlighted in green are shown in tables of article 2.

Table D : Annual impacts and costs of liver cancer in Women exposed to chlordecone in Guadeloupe after 2003

| **Parameters** | **source** | **unit** | **Group 1**  **mean estimate** | **Group 2**  **mean estimate** | **Group 3**  **mean estimate** | **Group 4**  **mean estimate** | **Group 5**  **mean estimate** | **∑** |
| --- | --- | --- | --- | --- | --- | --- | --- | --- |
| ERF (lifetime exposure, absolute risk) | cf. article 1 | (mg/kg/d)^-1^ | 2.69 | 2.69 | 2.69 | 2.69 | 2.69 | na |
| CW (change in weight unit | na | µg/mg | 1000 | 1000 | 1000 | 1000 | 1000 | na |
| CF_e/i_ | cf. article 1 | [µg/kg/d]/[µg/l_blood_] | 0.06 | 0.06 | 0.06 | 0.06 | 0.06 | na |
| Standard human lifetime | USEPA, 1988 [1] | yr | 70 | 70 | 70 | 70 | 70 | na |
| ERF (annual exposure, absolute risk) | equation 2 | (µg/l_blood_)^-1^ per year | 2.5E-06 | 2.5E-06 | 2.5E-06 | 2.5E-06 | 2.5E-06 | na |
| Exposure | Guldner, 2011 [2] | µg/l_blood_ | 0.182 |  | 0.291 | 0.650 | 4.168 | na |
| Excess Risk | equation 1 | (-) | 4.5E-07 |  | 7.2E-07 | 1.6E-06 | 1.0E-05 | na |
| Exposure distribution | Guldner, 2011 [2] | (-) | 38% |  | 12% | 25% | 25% | 100% |
| Population | INSEE 2002 | n | 90 998 |  | 28 736 | 59 867 | 59 867 | 239 470 |
| Impacts | equation 3 | case | 0.04 |  | 0.021 | 0.10 | 0.61 | 0.77 |
| DALY | WHO 2014a [3] | yr | 21.93 |  | 21.93 | 21.93 | 21.93 | na |
| VOLY | Quinet, 2013 [4] | €_2006_/yr | 111 327 |  | 111 327 | 111 327 | 111 327 | na |
| **Cost of Impacts (CI)** | **equation 5** | €_2006_ | **99 405** |  | **50 225** | **233 794** | **1 499 064** | **1 783 084** |
| Proportion of the total cost | Calculated | % | 6% | 0% | 3% | 13% | 84% | 100% |
|  |  |  | **Mean estimate** | **Low estimate** | **High estimate** |  |  |  |
| **CI without threshold** |  | €_2006_ | **1 882 489** | **1 264 797** | **3 853 219** |  |  |  |
| **F_thr_** | equation 4 | (-) | **26.1%** | **26.1%** | **26.1%** |  |  |  |
| **CI with threshold** | CI × F_thr_ | €_2006_ | **490 630** | **329 642** | **1 004 257** |  |  |  |

na: not appropriate. F_thr_: Fraction of collective exposure above threshold. ∑ = the sum of results of group 1 to group5. The results highlighted in green are shown in tables of article 2.

Table E : Annual impacts and costs of prostate cancer in Men > 44 yr exposed to chlordecone in Guadeloupe before 2003

| **Parameters** | **source** | **unit** | **Group 1**  **mean estimate** | **Group 2**  **mean estimate** | **Group 3**  **mean estimate** | **Group 4**  **mean estimate** | **Group 5**  **mean estimate** | **∑** |
| --- | --- | --- | --- | --- | --- | --- | --- | --- |
| ERF (lifetime exposure, absolute risk) | cf. article 1 | (µg/l_blood_)^-1^ | 0.00190 | 0.00190 | 0.00190 | 0.00190 | 0.00190 | na |
| Standard human lifetime | USEPA, 1988 | yr | 70 | 70 | 70 | 70 | 70 | na |
| ERF (annual exposure, absolute risk) | =ERF/standard human life | (µg/l_blood_)^-1^ per year | 2.71E-05 | 2.71E-05 | 2.71E-05 | 2.71E-05 | 2.71E-05 | na |
| Exposure | Guldner, 2011 [2] | µg/l_blood_ | 1.06 | 1.56 | 3.90 | 7.25 | 14.79 | na |
| Excess Risk | equation 1 | (-) | 2.89E-05 | 4.25E-05 | 1.06E-04 | 1.97E-04 | 4.01E-04 | na |
| Exposure distribution | Guldner, 2011 [2] | (-) | 9% | 16% | 25% | 25% | 25% | 100% |
| Population | INSEE 2002 | n | 6 531 | 11 611 | 18 141 | 18 141 | 18 141 | 72 566 |
| Impacts (New case) | equation 3 | case | 0.189 | 0.493 | 1.920 | 3.570 | 7.282 | 13.5 |
| Impacts (death) | WHO 2014 [3] | 1 death for 4,7 case | 0.039 | 0.103 | 0.401 | 0.745 | 1.519 | 2.8 |
| DALYs | Quinet, 2013 [4] | yr | 16.5 | 16.5 | 16.5 | 16.5 | 16.5 | na |
| VOLY | Guldner, 2011 [2] | €_2006_/yr | 111 327 | 111 327 | 111 327 | 111 327 | 111 327 | na |
| **Cost of Impacts (CI)** | **equation 5** | €_2006_ | **72 253** | **188 841** | **735 521** | **1 367 190** | **2 788 216** | **5 152 020** |
| Proportion of the total cost | Calculated | % | 1% | 4% | 14% | 27% | 54% | 100% |
|  |  |  | **Mean estimate** | **Low estimate** | **High estimate** |  |  |  |
| **CI without threshold** |  | €_2006_ | **5 152 020** | **1 405 096** | **10 571 677** |  |  |  |
| **F_thr_** | equation 4 | (-) | 65.1% | 65.1% | 65.1% |  |  |  |
| **CI with threshold** | CI × F_thr_ | €_2006_ | **3 352 710** | **914 375** | **6 879 586** |  |  |  |

na: not appropriate. F_thr_: Fraction of collective exposure above threshold. ∑ = the sum of results of group 1 to group5. The results highlighted in green are shown in tables of article 2.

Table F : Annual impacts and costs of prostate cancer in Men > 44 yr exposed to chlordecone in Guadeloupe after 2003

| **Parameters** | **Source** | **unit** | **Group 1**  **mean estimate** | **Group 2**  **mean estimate** | **Group 3**  **mean estimate** | **Group 4**  **mean estimate** | **Group 5**  **mean estimate** | **∑** |
| --- | --- | --- | --- | --- | --- | --- | --- | --- |
| ERF (lifetime exposure, absolute risk) | cf. article 1 | (µg/l_blood_)^-1^ | 0.00190 | 0.00190 | 0.00190 | 0.00190 | 0.00190 | na |
| Standard human lifetime | USEPA, 1988 | yr | 70 | 70 | 70 | 70 | 70 | na |
| ERF (annual exposure, absolute risk) | =ERF/standard human life | (µg/l_blood_)^-1^ per year | 2.71E-05 | 2.71E-05 | 2.71E-05 | 2.71E-05 | 2.71E-05 | na |
| Exposure | Guldner, 2011 [2] | µg/l_blood_ | 1.06 | 1.56 | 3.90 | 7.25 | 14.79 | na |
| Excess Risk | equation 1 | (-) | 2.89E-05 | 4.25E-05 | 1.06E-04 | 1.97E-04 | 4.01E-04 | na |
| Exposure distribution | Guldner, 2011 [2] | (-) | 9% | 16% | 25% | 25% | 25% | 100% |
| Population | INSEE 2002 | n | 10 114 | 8 898 | 19 012 | 19 012 | 19 012 | 76 049 |
| Impacts (New case) | equation 3 | case | 0.046 | 0.054 | 0.206 | 0.464 | 3.921 | 4.7 |
| Impacts (death) | WHO 2014 [3] | 1 death per 4,7 cases | 0.010 | 0.011 | 0.043 | 0.097 | 0.818 | 1.0 |
| DALYs | Quinet, 2013 [4] | yr | 16.5 | 16.5 | 16.5 | 16.5 | 16.5 | na |
| VOLY | Guldner, 2011 [2] | €_2006_/yr | 111 327 | 111 327 | 111 327 | 111 327 | 111 327 | na |
| **Cost of Impacts (CI)** | **equation 5** | €_2006_ | **17 588** | **20 722** | **79 067** | **177 897** | **1 501 451** | **1 796 724** |
| Proportion of the total cost | Calculated | % | 1% | 1% | 4% | 10% | 84% | 100% |
|  |  |  | **Mean estimate** | **Low estimate** | **High estimate** |  |  |  |
| **CI without threshold** |  | €_2006_ | **1 796 724** | **490 016** | **3 686 784** |  |  |  |
| **F_thr_** | equation 4 | (-) | 32.6% | 32.6% | 32.6% |  |  |  |
| **CI with threshold** | CI × F_thr_ | €_2006_ | **586 123** | **159 852** | **1 202 694** |  |  |  |

na: not appropriate. F_thr_: Fraction of cumulative exposure above threshold. ∑ = the sum of results of group 1 to group5. The results highlighted in green are shown in tables of article 2.

Table G : Annual impacts and costs of renal dysfunction in Women exposed to chlordecone in Guadeloupe before 2003

| **Parameters** | **Source** | **unit** | **Group 1**  **mean estimate** | **Group 2**  **mean estimate** | **Group 3**  **mean estimate** | **Group 4**  **mean estimate** | **Group 5**  **mean estimate** | **∑** |
| --- | --- | --- | --- | --- | --- | --- | --- | --- |
| ERF (lifetime exposure, absolute risk) | cf. article 1 | (mg/kg/d)^-1^ | 0.0022 | 0.0022 | 0.0022 | 0.0022 | 0.0022 | na |
| CW (change in weight unit) | Na | µg/mg | 1000 | 1000 | 1000 | 1000 | 1000 | na |
| CF_e/i_ | cf. article 1 | [µg/kg/d]/[µg/l_blood_] | **0.06** | **0.06** | **0.06** | **0.06** | **0.06** | na |
| ERF (annual exposure, absolute risk) | equation 2 | (µg/l_blood_)^-1^ | 1.41E-07 | 1.4E-07 | 1.4E-07 | 1.4E-07 | 1.4E-07 | na |
| Exposure | Guldner, 2011 [2] | µg/l_blood_ | 0.3205 | 0.6202 | 1.7000 | 3.0500 | 8.0461 | na |
| Excess Risk | equation 1 | (-) | 4.5E-08 | 8.7E-08 | 2.4E-07 | 4.3E-07 | 1.1E-06 | na |
| Exposure distribution | Guldner, 2011 [2] | (-) | 13% | 12% | 25% | 25% | 25% | na |
| population | INSEE 2002 | n | 29 564 | 27 290 | 56 854 | 56 854 | 56 854 | 227 417 |
| Impacts (I) | equation 3 | case | 0.00133 | 0.00238 | 0.01361 | 0.02442 | 0.06441 | 0.106 |
| DALYs | WHO 2014 [3] | yr | 26.07 | 26.07 | 26.07 | 26.07 | 26.07 | na |
| VOLY | Quinet, 2013 [4] | €_2006_/yr | 111 327 | 111 327 | 111 327 | 111 327 | 111 327 | na |
| **Cost of Impacts (CI)** | **equation 5** | €_2006_ | **3 873** | **6 917** | **39 503** | **70 873** | **186 968** | 308 134 |
| Proportion of the total cost | Calculated | % | 1% | 2% | 13% | 23% | 61% | 100% |
|  |  |  | **Mean estimate** | **Low estimate** | **High estimate** |  |  |  |
| **CI without threshold** |  | €_2006_ | **308 134** | **26 643** | **769 234** |  |  |  |
| **F_thr_** | equation 4 | (-) | 34.5% | 34.5% | 34.5% |  |  |  |
| **CI with threshold** | CI × Fthr | €_2006_ | **106 418** | **9 201** | **265 665** |  |  |  |

na: not appropriate. F_thr_: Fraction of cumulative exposure above threshold. ∑ = the sum of results of group 1 to group5. The results highlighted in green are shown in tables of article 2.

Table H : Annual impacts and costs of renal dysfunction in Women exposed to chlordecone in Guadeloupe after 2003

| **Parameters** | **source** | **unit** | **Group 1**  **mean estimate** | **Group 2**  **mean estimate** | **Group 3**  **mean estimate** | **Group 4**  **mean estimate** | **Group 5**  **mean estimate** | **∑** |
| --- | --- | --- | --- | --- | --- | --- | --- | --- |
| ERF (lifetime exposure, absolute risk) | cf. article 1 | (mg/kg/d)^-1^ | 0.0022 | 0.0022 | 0.0022 | 0.0022 | 0.0022 | na |
| CW (change in weight unit) | na | µg/mg | 1000 | 1000 | 1000 | 1000 | 1000 | na |
| CF_e/i_ | cf. article 1 | [µg/kg/d]/[µg/l_blood_] | **0.06** | **0.06** | **0.06** | **0.06** | **0.06** | na |
| ERF (annual exposure, absolute risk) | equation 2 | (µg/l_blood_)^-1^ | 1.41E-07 | 1.41E-07 | 1.4E-07 | 1.4E-07 | 1.4E-07 | na |
| Exposure | Guldner, 2011 [2] | µg/l_blood_ | 0.1818 | 0.1818 | 0.2909 | 0.6500 | 4.1677 | na |
| Excess Risk | equation 1 | (-) | 2.6E-08 | 2.6E-08 | 4.1E-08 | 9.2E-08 | 5.9E-07 | na |
| Exposure distribution | Guldner, 2011 [2] | (-) | 38% | 0% | 12% | 25% | 25% | 100% |
| population | INSEE 2002 | n | 90 998 |  | 28 736 | 59 867 | 59 867 | 239 470 |
| Impacts (I) | equation 3 | case | 0.00233 |  | 0.00118 | 0.00548 | 0.03513 | 0.0441 |
| DALYs | WHO 2014 [3] | yr | 26.07 |  | 26.07 | 26.07 | 26.07 | na |
| **VOLY** | Quinet, 2013 [4] | €_2006_/yr | 111 327 |  | 111 327 | 111 327 | 111 327 | na |
| **Cost of Impacts (CI)** | **equation 5** | €_2006_ | **6 762** |  | **3 417** | **15 905** | **101 978** | **128 062** |
| Proportion of the total cost | Calculated | % | 5% | 0% | 3% | 12% | 80% | 100% |
|  |  |  | **Mean estimate** | **Low estimate** | **High estimate** |  |  |  |
| **CI without threshold** |  | €_2006_ | **128 062** | **11 073** | **319 698** |  |  |  |
| **F_thr_** | equation 4 | (-) | 26.1% | 26.1% | 26.1% |  |  |  |
| **CI with threshold** | CI × F_thr_ | €_2006_ | **33 377** | **2 886** | **83 322** |  |  |  |

na: not appropriate. F_thr_: Fraction of cumulative exposure above threshold. ∑ = the sum of results of group 1 to group5. The results highlighted in green are shown in tables of article 3.

Table I : Annual impacts and costs of cognitive development in boys born to Women exposed to chlordecone in Guadeloupe before 2003

| **Parameters** | **source** | **unit** | **Group 1**  **mean estimate** | **Group 2**  **mean estimate** | **Group 3**  **mean estimate** | **Group 4**  **mean estimate** | **Group 5**  **mean estimate** | **∑** |
| --- | --- | --- | --- | --- | --- | --- | --- | --- |
| ERF | Boucher 2013 [6] | IQ-lost per µg/l_cord-blood_ | 0.320 | 0.320 | 0.320 | 0.320 | 0.320 | na |
| Exposure (blood cord newborn) | Gulner 2011 [2] | µg/l_cord-blood_ | 0.394 | 0.394 | 0.547 | 0.950 | 2.107 | na |
| Excess Risk | equation 1 | IQ-lost | 0.126 | 0.126 | 0.175 | 0.304 | 0.674 | na |
| Exposure distribution | Gulner 2011 [2] | (-) | 39% | 0% | 11% | 25% | 25% | 100% |
| Population (newborn boy) | [INSEE](http://www.insee.fr/fr/themes/document.asp?ref_id=12122) 2002 | (-) | 1 461 |  | 412 | 937 | 937 | **3 747** |
| Impacts | equation 3 | IQ loss per yr | 184 |  | 72 | 285 | 632 | 1 173 |
| Cost / QI point | Pichery, 2011 [7] | €_2006_/IQ | 16 837 |  | 16 837 | 16 837 | 16 837 | na |
| **Cost of impacts** | **equation 5** | €_2006_ | **3 100 279** |  | **1 214 488** | **4 794 843** | **10 635 117** | **19 744 726** |
| Proportion of the total cost | Calculated | % | 16% | 0% | 6% | 24% | 54% | 100% |
|  |  |  | **Mean estimate** | **Low estimate** | **High estimate** |  |  |  |
| **CI without threshold** |  | €_2006_ | **19 744 726** | **3 948 945** | **39 489 453** |  |  |  |
| **F_thr_** | equation 4 | (-) | **85.7%** | **85.7%** | **85.7%** |  |  |  |
| **CI with threshold** | CI × Fthr | €_2006_ | **16 922 241** | **3 384 448** | **33 844 482** |  |  |  |

na: not appropriate. F_thr_: Fraction of cumulative exposure above threshold. ∑ = the sum of results of group 1 to group5. The results highlighted in green are shown in tables of article 2.

Table J : Annual impacts and costs of cognitive development in boys born to Women exposed to chlordecone in Guadeloupe after 2003

| **Parameters** | **source** | **unit** | **Group 1**  **mean estimate** | **Group 2**  **mean estimate** | **Group 3**  **mean estimate** | **Group 4**  **mean estimate** | **Group 5**  **mean estimate** | **∑** |
| --- | --- | --- | --- | --- | --- | --- | --- | --- |
| FER | Boucher 2013 [6] | IQ-lost per µg/l_cord-blood_ | 0.320 | 0.320 | 0.320 | 0.320 | 0.320 | na |
| Exposure (blood cord new born) | Gulner 2011 [2] | µg/l_cord-blood_ | 0.394 | 0.394 | 0.547 | 0.950 | 2.107 | na |
| Risk | equation 1 | IQ-lost | 0.126 | 0.126 | 0.175 | 0.304 | 0.674 | na |
| Exposure distribution | Gulner 2011 [2] | (-) | 39% | 0% | 0% | 25% | 25% | 100% |
| Population (new-born boy) | [INSEE](http://www.insee.fr/fr/themes/document.asp?ref_id=12122) 2002 | (-) | 2829 |  |  | 118 | 982 | **3929** |
| Impacts | equation 3 | IQ loss per yr | 170 |  |  | 9 | 824 | 1003 |
| Cost / QI point | Pichery, 2011 [7] | €_2006_/IQ | 16837 |  |  | 16837 | 16837 | na |
| **Cost of impacts** | **equation 5** | €_2006_ | **2 862 084** |  |  | **154 876** | **13 869 775** | **16 886 735** |
| Proportion of the total cost | Calculated | % | 17% | 0% | 0% | 1% | 82% | 100% |
|  |  |  | **Mean estimate** | **Low estimate** | **High estimate** |  |  |  |
| **CI without threshold** |  | €_2006_ | **16 886 735** | **3 377 347** | **33 773 471** |  |  |  |
| **F_thr_** | equation 4 | (-) | **63.83%** | **63.83%** | **63.83%** |  |  |  |
| **CI with threshold** | CI × F_thr_ | €_2006_ | **10 778 483** | **2 155 697** | **21 556 966** |  |  |  |

na: not appropriate. F_thr_: Fraction of cumulative exposure above threshold. ∑ = the sum of results of group 1 to group 5. The results highlighted in green are shown in tables of article 2.

**References**

1. USEPA. Recommendations for and Documentation of Biological Values for Use in Risk Assessment. Washington DC: United States Environnemental Agency1988 February. Report No.: EPA/600/6-87/008.

2. Guldner L, Seurin S, Héraud F, Multignier L. Exposition de la population antillaise au chlordécone. BEH. 2011;Numéro thématique – Chlordécone aux Antilles : bilan actualisé des risques sanitaires(3-4-5):25-8. <http://www.invs.sante.fr/beh/2011/03_04_05/beh_03_04_05.pdf>

3. WHO. WHO global health estimates 2014 summary tables: DALY by cause, age and sex, 2000-2012. Geneva: Department of Health Statistics and Information Systems Whorld Health Organisation2014a June. And : WHO. WHO global health estimates 2014 summary tables: Deaths by cause, age and sex, 2000-2012. Geneva: Department of Health Statistics and Information Systems Whorld Health Organisation2014b June.

4. Quinet E. L’évaluation socioéconomique des investissements publics. Tome 1, rapport final. (Socio-economic evaluation of public investments). Paris: Commissariat Général à la stratégie et à la prospective, 2013.

5. Joachim C, Macni J, Véronique-Baudin J, Piccotti C, Escarmant P. Epidémiologie du cancer de la prostate aux Antilles-Guyane : données des registres généraux des cancers. BVS. 2013;8-9:3-5.

6. Boucher O, Simard MN, Muckle G, Rouget F, Kadhel P, Bataille H et al. Exposure to an organochlorine pesticide (chlordecone) and development of 18-month-old infants. Neurotoxicology. 2013;35C:162-8. doi:10.1016/j.neuro.2013.01.007.

7. Pichery C, Bellanger M, Zmirou-Navier D, Glorennec P, Hartemann P, Grandjean P. Childhood lead exposure in France: benefit estimation and partial cost-benefit analysis of lead hazard control. Environmental health : a global access science source. 2011;10:44. doi:10.1186/1476-069x-10-44.
